# Correction: Mathematical bridge between epidemiological and molecular data on cancer and beyond

**DOI:** 10.1371/journal.pone.0336448

**Published:** 2025-11-07

**Authors:** The PLOS One Staff

## Notice of Republication

This article was republished on July 28, 2025, because the PDF and HTML versions did not match. In the html the following text was removed because it was duplicated:

“At least six different mathematical models of cancer and their countless variations and combinations have been published to date in the scientific literature that reasonably explain epidemiological prediction of multi-step carcinogenesis.” The publisher apologizes for the errors. Please download this article again to view the correct version. The originally published, uncorrected article and the republished, corrected articles are provided here for reference.

## Supporting information

S1 File Originally published, uncorrected article. (PDF)

S2 File Republished, corrected article. (PDF)
