# Supplementary material for: Mathematical bridge between epidemiological and molecular data on cancer and beyond
Source: PLoS One. 2025 Jul 28;20(7):e0328401. doi: 10.1371/journal.pone.0328401 (PMC12303294; doi:10.1371/journal.pone.0328401)
Supplement: S1 — (PDF) [file pone.0328401.s001.pdf]

Supporting information

Derivations

Standard model of multistep carcinogenesis refers to the mathematical as well as statistical framework which allows us to simulate, test and predict the number of rate-limiting steps for a given cancer in order to gain mechanistic insight into carcinogenesis through communication between epidemiological and molecular data. We will derive the standard model in the following steps.

1. Derivation of the probability of a given number of sequential rate-limiting events to occur within a given duration of time.
2. Derivation of the probability of r-th rate-limiting events to occur in an infinitesimal time interval after $r-1$ such events have already occurred.
3. Evaluation of the function that takes time as input and gives probability as output. Here, the probability means the chance that an individual of the age group denoted by the time does not manifest a cancer yet, all other quantities being constant.
4. Derivation of the formula for age-specific incidence rate of cancer.

Step 1

If $r-1$ rate-limiting events occur with $k_1, k_2, \ldots, k_{r-1}$ probability per unit of time then the probability of each of those occurring in course of time t would be $k_1 t, k_2 t, \ldots, k_{r-1} t$, respectively, provided that the values of $k_1, k_2, \ldots, k_{r-1}$ are small enough to allow the probabilities $k_1 t, k_2 t, \ldots, k_{r-1} t$ to scale for a value of t large enough to accommodate typical average human lifespan (Assumption 2). The probability of $r-1$ rate-limiting events to occur in any order during a period of t would thus be,

$$K_{\text{unordered}} = (k_1 t)(k_2 t) \ldots (k_{r-1} t) = (k_1 k_2 \ldots k_{r-1}) t^{r-1} \quad \text{... Equation 1}$$

Since the sequence of mutation has to be ordered, we are interested in the probability of only one out of $(r-1)!$ possible arrangements of rate-limiting event (Assumption 3). Therefore the probability of $r-1$ mutations to occur in a given order during a period of t would be,

$$K_{\text{ordered}} = \frac{K_{\text{unordered}}}{(r-1)!} = \frac{(k_1 k_2 \ldots k_{r-1}) t^{r-1}}{(r-1)!} \quad \text{... Equation 2}$$

Step 2

If the r-th step, the final rate-limiting event required for cancer manifestation, occurs with a probability k per unit of time within a short interval $(t, t+dt)$, then the actual probability of that event would be $k \cdot dt$.

Let the aggregated ordered rate of occurrence of events per unit of time or the probability of all r events to occur in a specific order within the given interval of time,

$$k = \frac{k_1 k_2 \ldots k_{r-1} k_r}{(r-1)!} \quad \text{... Equation 3}$$

The total probability of $(r-1)$ rate-limiting events to occur in a given order during t followed by the occurrence of r-th event during dt would thus be,

$$K = K_{\text{ordered}} k_r dt = \left(\frac{k_1 k_2 \ldots k_{r-1} \cdot t^{r-1}}{(r-1)!} \right) k_r \cdot dt = k \cdot t^{r-1} \cdot dt \quad \text{... Equation 4}$$

Step 3

Let $P(t)$ be the probability that an individual of age t has not manifested a given type of cancer yet. Then the probability of randomly choosing an individual of age t who eventually develops cancer after a short interval of dt would be,

$$KP(t) = P(t) k \cdot t^{r-1} \cdot dt \quad \text{... Equation 5}$$

This quantity is equivalent to the frequency of individuals of age t developing cancer after they have reached that age and before they reach the age of $(t+1)$, which is by definition, age specific incidence rate at age t. We proceed by eliminating the dt term to obtain a mathematically rigorous form of the age-specific incidence rate.

If an individual is chosen from any age group in the range 0 to t, the probability of developing cancer is represented by the integral over time:

$$\sum_{0}^{t} KP(t) \approx \int_0^t P(\tau) k \tau^{r-1} \, d\tau \quad \text{... Equation 6}$$

Please note that their individual probabilities are disjoint events. The integral can also be thought of as a cumulative frequency of individuals between ages 0 and t who developed cancer while remaining at their respective age groups. If a population begins with a probability of $P(0)$ which denotes the individuals at age 0 without the manifestations of cancer, and then proceeds up to age t while 'losing' individuals who succumb to cancer at each successive age groups, then

$$P(t) = P(0) - \int_0^t P(\tau) k\tau^{r-1} d\tau \quad \text{... Equation 7}$$

Since $P(0)$ is a constant term by virtue of being the 0th term of a function, differentiating both sides with respect to dt gives us,

$$\frac{d}{dt}P(t) = 0 - P(t)k\,t^{r-1} \Rightarrow \frac{dP(t)}{P(t)} = -k\,t^{r-1}\,dt$$

Integrating both sides,

$$\ln P(t) = -\frac{k\,t^r}{r} + C_0 \Rightarrow P(t) = e^{-\frac{k\,t^r}{r+C_0}} = C_1 e^{-\frac{k\,t^r}{r}}$$

$$\text{[where } C_0, C_1 \text{ are constants; let } C_1 = e^{C_0}\text{]}$$

Since at $t = 0$, $P(0) = C_1$,

$$P(t) = P(0)e^{-\frac{k\,t^r}{r}} \quad \text{... Equation 8}$$

Step 4

$P(t)$ is a cumulative distribution function (CDF) that expresses the total probability of not developing cancer in the age range of 0 to t. It can be thought of as a survival function in a sense that, it denotes the portion of the population at age t that has 'survived' by not developing cancer yet. More rigorously, if we take a CDF, $F(t)$ to indicate the probability of developing cancer in the age range of 0 to t then by definition of survival function,

$$P(t) = 1 - F(t) \quad \text{... Equation 9}$$

We want to determine age specific incidence rate, the probability of developing cancer at any given short interval $(t, t + dt)$ which is nothing but the hazard function of $F(t)$, since hazard function is defined as the probability of an event to occur in a short interval. Since the probability density function (PDF) of $F(t)$ is given by the derivative of $F(t)$ with respect to dt and hazard function is the ratio of PDF to survival function, age specific incidence rate at age t would be,

$$I(t) = \frac{\frac{d}{dt}F(t)}{1 - F(t)} = \frac{\frac{d}{dt}(1 - P(t))}{P(t)} = \frac{P(0)kt^{r-1}e^{-kt^r/r}}{P(0)e^{-kt^r/r}} = kt^{r-1} \quad \text{... Equation 10}$$

This nice and simple equation can be reframed using logarithm as,

$$\ln I(t) = (r-1)\ln t + \ln k \quad \text{... Equation 11}$$

Derivation of the non-linear models from heterogeneity assumption

If we replace $k = k_p t^p + k_q t^q$ in Equation 4 with the following assumption, k_p and k_q are the respective probabilities of a rate-limiting event to occur with the rate t^p and t^q where p and q are real numbers, then the equation becomes,

$$K = (k_p t^p + k_q t^q)t^{r-1}dt = k_p t^{r+p-1}dt + k_q t^{r+q-1}dt \quad \text{... Equation 12}$$

The rationale behind this assumption is the diversity of factors and mechanisms underlying the heterogeneous groups of disorders clumped under a single label, e.g. leukemia. The simplest form that could accommodate such a situation is a two-factor model which is incorporated in the assumption above. If we follow through the calculations according to the derivation of the standard model, then this new assumption ultimately leads to,

$$I(t) = k_p t^{r+p-1} + k_q t^{r+q-1} = k_p t^{r+p-1}\left(1 + \frac{k_q}{k_p}t^{q-p}\right) \quad \text{... Equation 13}$$

Taking log on each side,

$$\ln I(t) = \ln k_p + (r+p-1)\ln t + \ln\left(1 + \frac{k_q}{k_p}t^{q-p}\right) \quad \text{... Equation 14}$$

This is the general form of the equation for log-log plots with upwards concavity. If instead, the initial two-factor term (Equation 12) was assumed to be $k = k_p t^p - k_q t^q$ then the ultimate log-log form would become,

$$\ln I(t) = \ln k_p + (r+p-1)\ln t + \ln\left(1 - \frac{k_q}{k_p}t^{q-p}\right) \quad \text{... Equation 15}$$

This is the general form of the equation for log-log plots with upwards convexity. An additional assumption of $k_q \leq k_p$ would allow for $0 \leq \frac{k_q}{k_p} \leq 1$ to be true while validating the models using real world data. This would help normalize the dataset without compromising the rigor of the method. Also, putting $k_q = 0$ regresses the equations back to the standard linear form.

Derivation of the non-linear models from age-related effect assumption

If we take into consideration that some factors accelerate or decelerate the rate of tumor progression with increasing age then we could formulate an alternate set of assumptions for non-linear models.

If we add a factor $k_a t^a$ to account for the age-related acceleration in Equation 4 with the following assumption, k_a is the probability of a rate-limiting event to occur with the rate t^a a is a real number, then the equation becomes,

$$K = kt^{r-1}dt + k_a t^a dt \quad \text{... Equation 16}$$

If we follow through the calculations according to the derivation of the standard model, then this new assumption ultimately leads to,

$$I(t) = kt^{r-1} + k_a t^a = kt^{r-1}\left(1 + \frac{k_a}{k}t^{a-r+1}\right) \quad \text{... Equation 17}$$

Taking log on each side,

$$\ln I(t) = \ln k + (r-1)\ln t + \ln\left(1 + \frac{k_a}{k}t^{a-r+1}\right) \quad \text{... Equation 18}$$

This is the general form of the equation for log-log plots with upwards concavity. Similarly, subtracting an age-related deceleration factor $k_d t^d$ from Equation 4 would ultimately result in,

$$\ln I(t) = \ln k + (r-1)\ln t + \ln\left(1 - \frac{k_d}{k}t^{d-r+1}\right) \quad \text{... Equation 19}$$

This is the general form of the equation for log-log plots with upwards convexity. Since the terms k, k_a, and k_d are probabilities, the fractional terms inside the third log term of Equation 18 and Equation 19 would always be within 0 and 1, inclusive. Also, if there is no age-related acceleration ($k_a = 0$) or deceleration ($k_d = 0$) or if they are small enough to be ignored then the non-linear form regresses to the standard linear form.

Extended Data Figures

For all the graphs in appendix B, X axis denotes the age groups in log scale and Y axis denotes the age specific incidence rates in log scale.

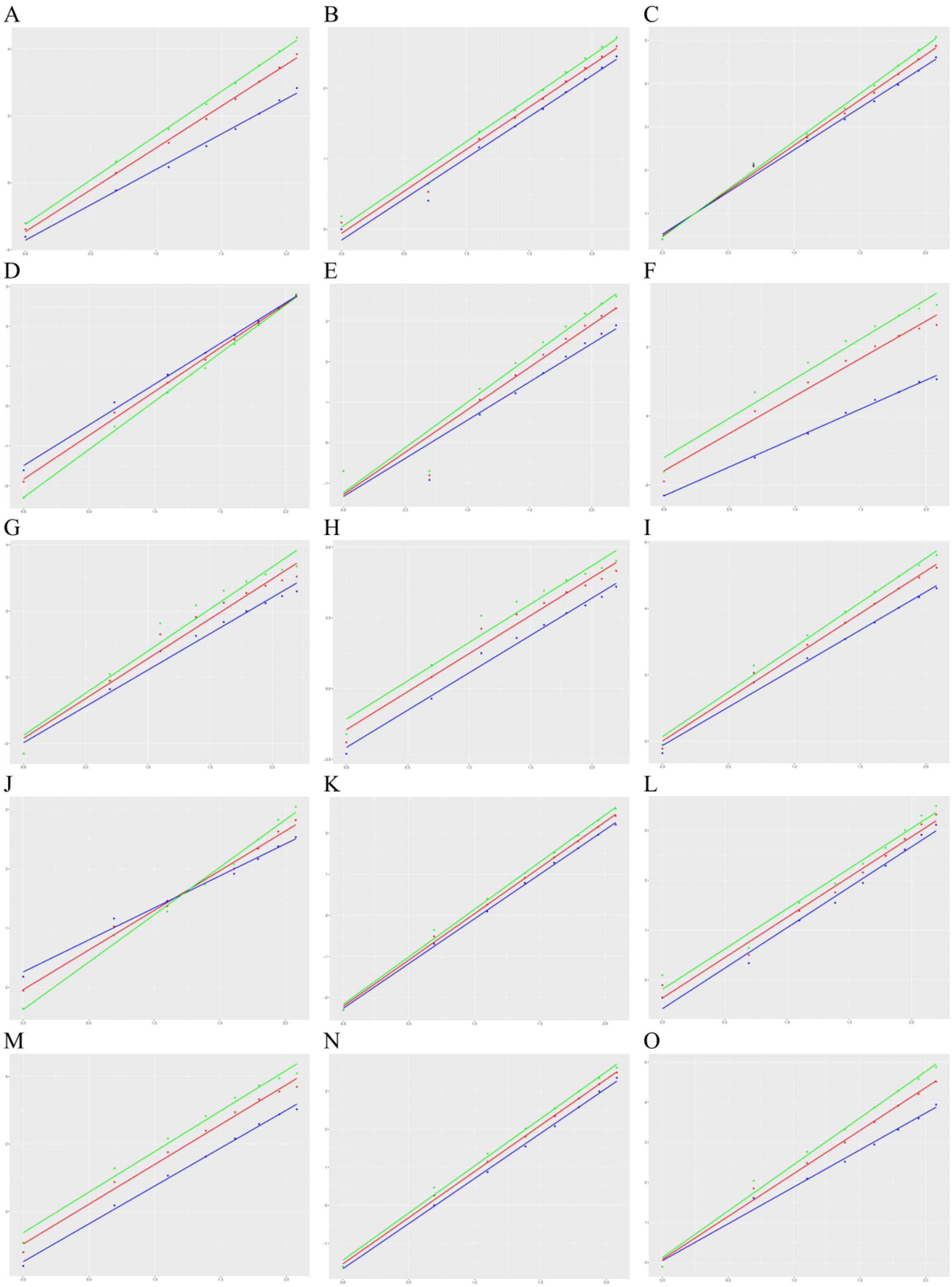

Figure 1. Cancer categories best fitting the linear log-log model. The horizontal and vertical axes show age class and incidence, respectively, both in log scale. Blue, green, and red lines and dots represent the regression lines and data points for female, male, and both sexes, respectively. A, bladder cancer; B, cancer of brain and nervous system; C, colorectal cancer; D, gallbladder cancer; E, kidney cancer; F, cancer of larynx; G, cancer of lip and oral cavity; H, liver cancer; I, lung cancer; J, skin melanoma; K, multiple myeloma; L, non-Hodgkin lymphoma; M, cancer of esophagus; N, pancreatic cancer; O, stomach cancer.

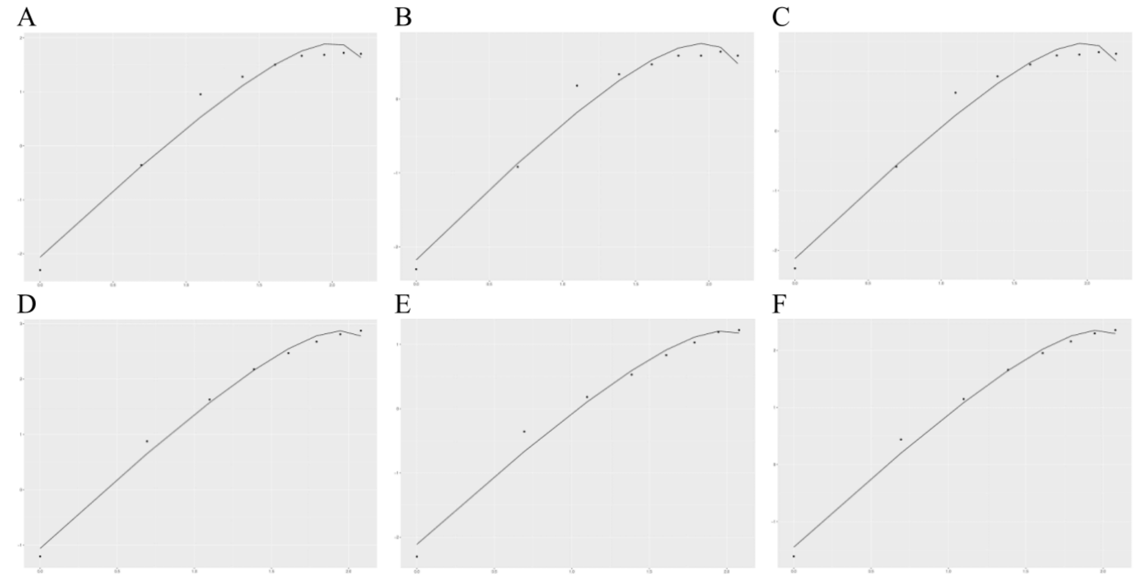

Figure 2. Cancer categories best fitting the convex upwards model. The horizontal and vertical axes show age class and incidence, respectively, both in log scale. A, cancer of nasopharynx in male; B, cancer of nasopharynx in female; C, cancer of nasopharynx in both sexes; D, cancer of other parts of the pharynx in male; E, cancer of other parts of the pharynx in female; F, cancer of other parts of the pharynx in both sexes.

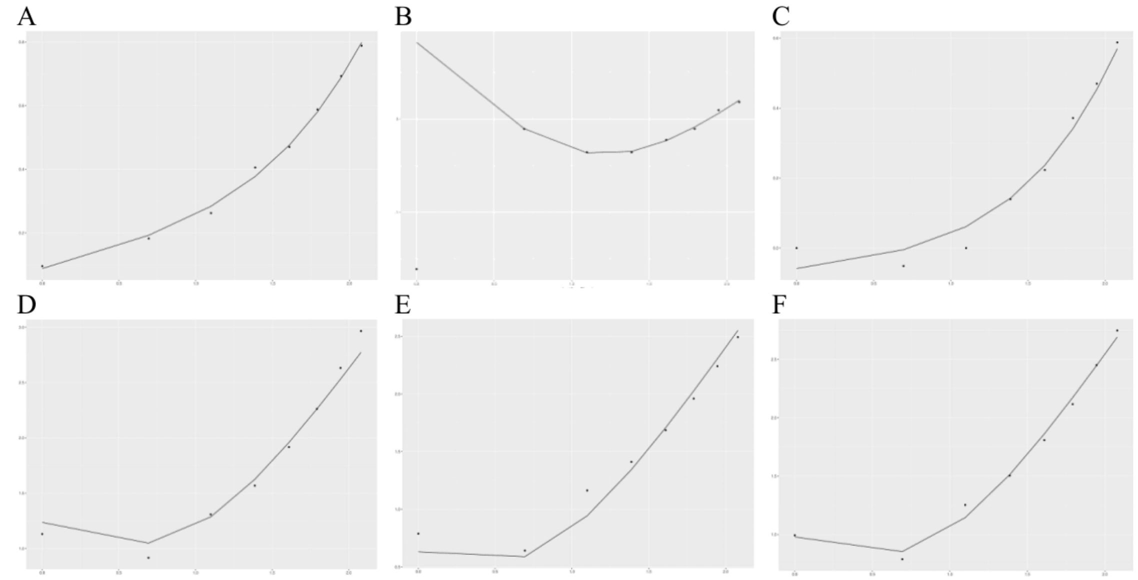

Figure 3. Cancer categories best fitting the concave upwards model. The horizontal and vertical axes show age class and incidence, respectively, both in log scale. A, Hodgkin lymphoma in male; B, Hodgkin lymphoma in female; C, Hodgkin lymphoma in both sexes; D, leukemias in male; E, leukemias in female; F, leukemias in both sexes.

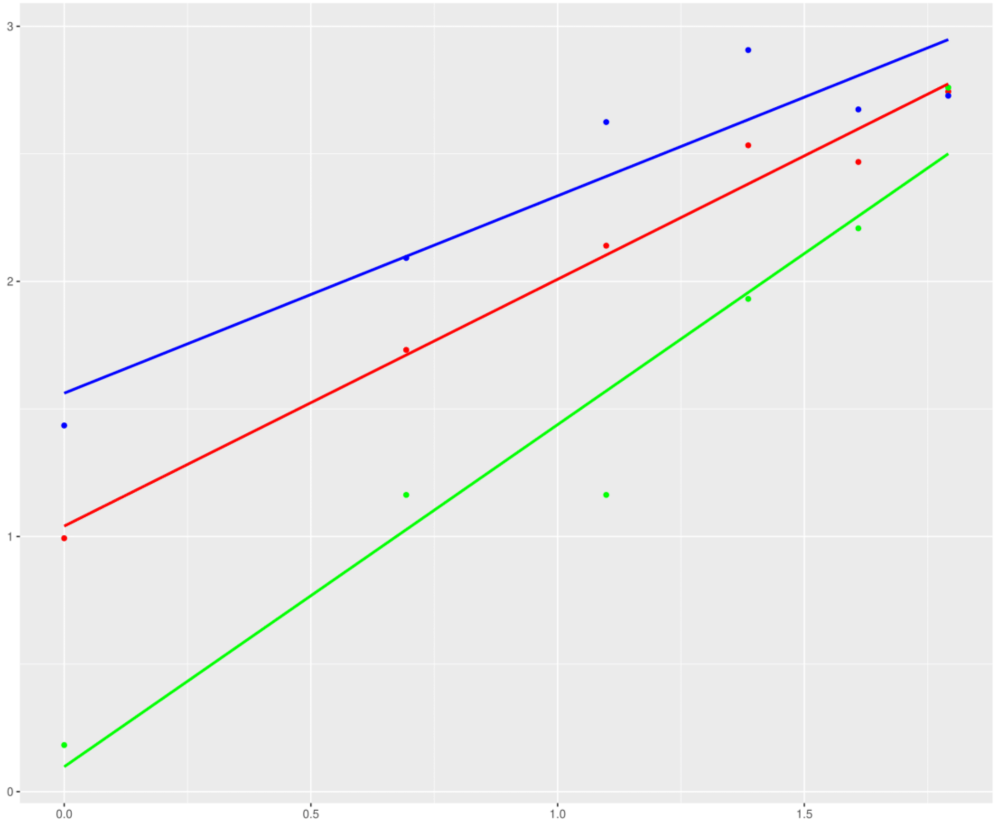

Figure 4. The linear log-log model has been extended to predict genetic and pathophysiological rate-limiting events in rheumatoid arthritis, a non-neoplastic condition. The horizontal and vertical axes show age class and incidence, respectively, both in log scale. Blue, green, and red lines and dots represent the regression lines and data points for female, male, and both sexes, respectively. Age groups of 21-30, 31-40, 41-50, 51-60, 61-70, and >70 years have been denoted by the age classes 1-6, respectively. The equations of the regression line for male: log (incidence) = 0.0975 + 1.3411 × log (age class), R^2 = 0.9377; for female: log (incidence) = 1.5619 + 0.7735 × log (age class), R^2 = 0.8668; for both sexes: log (incidence) = 1.0407 + 0.9677 × log (age class), R^2 = 0.9787.
