# Supplementary material for: Mathematical bridge between epidemiological and molecular data on cancer and beyond
Source: PLoS One. 2025 Jul 28;20(7):e0328401. doi: 10.1371/journal.pone.0328401 (PMC12303294; doi:10.1371/journal.pone.0328401)
Supplement: Attachment — Submitted filename: ResponseReviewers.pdf [file pone.0328401.s003.pdf]

# Response to Reviewers

Dear Editor and Reviewers,

We are pleased that our investigation was considered to have merit by the reviewers and editors. PLOS ONE has invited us to submit a revised version of the manuscript that addresses the points raised during the review process. We thank the anonymous reviewer for their careful appraisal and valuable suggestions. We have addressed all of these suggestions and substantially revised the manuscript accordingly. Below, we provide a point-by-point response to their recommendations. The reviewers' statements are printed in gray; our responses follow in black, with changes marked blue and the associated page and line of the manuscript before revision indicated.

# Editor Comments

**Editor Comment 1** — Journal requirements:

When submitting your revision, we need you to address these additional requirements.

1. Please ensure that your manuscript meets *PLOS ONE*'s style requirements, including those for file naming. The *PLOS ONE* style templates can be found at:
   - https://journals.plos.org/plosone/s/file?id=wjVg/PLOSOne_formatting_sample_main_body.pdf
   - https://journals.plos.org/plosone/s/file?id=ba62/PLOSOne_formatting_sample_title_authors_affiliations.pdf

**Response**: Thank you for your comment. We have used the PLOS ONE's LaTeX template to meet with the style requirements.

**Editor Comment 2** — We note that your Data Availability Statement is currently as follows: [All relevant data are within the manuscript and its supporting information files.]

Please confirm at this time whether or not your submission contains all raw data required to replicate the results of your study. Authors must share the "minimal data set" for their submission. PLOS defines the minimal data set to consist of the data required to replicate all study findings reported in the article, as well as related metadata and methods (https://journals.plos.org/plosone/s/data-availability#loc-minimal-data-set-definition).

For example, authors should submit the following data:

- The values behind the means, standard deviations and other measures reported;
- The values used to build graphs;
- The points extracted from images for analysis.

Authors do not need to submit their entire data set if only a portion of the data was used in the reported study.

If your submission does not contain these data, please either upload them as Supporting Information files or deposit them to a stable, public repository and provide us with the relevant URLs, DOIs, or accession numbers. For a list of recommended repositories, please see `https://journals.plos.org/plosone/s/recommended-repositories`.

If there are ethical or legal restrictions on sharing a de-identified data set, please explain them in detail (e.g., data contain potentially sensitive information, data are owned by a third-party organization, etc.) and who has imposed them (e.g., an ethics committee). Please also provide contact information for a data access committee, ethics committee, or other institutional body to which data requests may be sent. If data are owned by a third party, please indicate how others may request data access.

**Response**: Thank you for highlighting the importance of ensuring full data transparency and reproducibility. In response, we have now included a new section titled **Data and Code Availability** in our revised manuscript. To address this concern, we have added the following section in our manuscript:

## Data and code Availability

The code for reproducing this paper is available in Github. The dataset for cancer incidence has been sourced from CI5 (Cancer Incidence in Five Continents) of GLOBOCAN.

**Editor Comment 3** — Please update your submission to use the *PLOS* LaTeX template. The template and more information on our requirements for LaTeX submissions can be found at:

`http://journals.plos.org/plosone/s/latex`

**Response**: We have updated our submission to use the *PLOS* LaTeX template.

**Editor Comment 4** — 4. Please include a separate caption for each figure in your manuscript.

**Response**: Thank you for your guidance. We fully understand the importance of having separate captions for each figure to ensure clarity and accessibility.

However, our manuscript contains a total of 28 figures, corresponding to cancer-specific incidence curves for multiple populations. To maintain readability and to avoid excessive length, we have grouped these figures based on the three mathematical models developed in the paper—namely, the linear log-log model, convex upwards model, and concave upwards model and also a special case: non-neoplastic condition. Each group of figures is accompanied by a unified caption that explains the context and relevance of the model-specific validation. This approach was chosen to help readers focus on the structural interpretation and numerical validation provided by the model groupings.

We hope this structured grouping is acceptable.

5. We note you have included a table to which you do not refer in the text of your manuscript. Please ensure that you refer to Table 1-3 in your text; if accepted, production will need this reference to link the reader to the Table.

**Editor Comment 5** — We have included the following paragraph in the **Model Validation** section so that the tables are referred in the text.

**Changes**: " For the linear log-log model, we calculated the slope, intercept, and $R^2$ values for the data-fitted graphs.The results of our regression analysis are summarized in **Table**1, which lists the cancer categories best fitting the linear log-log model; **Table**2, which presents those best described by the convex upwards model; and **Table**3, which includes those fitting the concave upwards model. These tables provide the estimated parameters and $R^2$ values for each model, for the different genders."

---

## Reviewer 1

Please correct word processing errors. I would suggest going through the paper again to catch the odd missing word and some word processing errors.

**Reviewer Statement 1.1** — Incorrect list numbering 8. in section 4.2.3, page 11.

**Response**: Thank you for pointing out the list numbering inconsistency in Section 4.2.3. To address this, we revised the text to read: But from the molecular data on tumor progression, two inferences can be incorporated as extended assumptions when assumption 7 does not hold (continuing from **Assumptions 1-7**) :

8. Gender-associated differences in the slopes for a given cancer may be attributed to different values of the exponent of the aforementioned exponential mutation rate between genders and not to the possibility of having different numbers of rate-limiting events for the same cancer in males and females.

9. Assumption 8 may be repurposed and incorporated to quantify geographical and/or ethnic as well as environmental and lifestyle-related differences of cancer progression.

This change clarifies that the following assumptions 8 and 9 directly build upon assumptions 1–7.

This adjustment clarifies that the subsequent assumptions build upon assumptions 1–7. The updated numbering and context are now consistent in this section and throughout the manuscript.

**Changes**: Adding "(continuing from **Assumptions 1-7**) :" to the end of the sentence: But from the molecular data on tumor progression, two inferences can be incorporated as extended assumptions when assumption 7 does not hold

**Reviewer Statement 1.2** — Please remove paragraph indents after formulas (page 3, after Eq. 1; page 6, after Table 3; page 9, after Eq. 8 and Eq. 9; page 10, after Eq. 11; page 11, after Eq. 17 and Eq. 19).

**Response**: Thank you for this suggestion. We have carefully reviewed and removed the paragraph indents following the specified formulas and table to maintain consistency in formatting. The manuscript now follows a uniform style throughout these sections.

**Reviewer Statement 1.3** — Lack of explanations of symbols in Eq. 3, Eq. 4, Eq. 6, Eq. 7.

**Response**: Thank you for highlighting this issue. We have added concise explanations for all symbols used in Equations 3, 4, 6, and 7 in the corresponding sections of the manuscript. These clarifications

provide readers with clear definitions of the parameters and enhance the overall readability of the derivations.

**Changes**: **Convex upwards model from heterogeneity assumption**

$$\ln I(t) = \ln k_p + (r + p - 1) \ln t + \ln \left(1 - \frac{k_q}{k_p} t^{q-p}\right)$$

where:

- $k_p, k_q$: Probabilities per unit time for two distinct population subgroups or mechanistic pathways that contribute to the overall disease process.
- $p, q$: Exponents indicating how the risk of each subgroup scales with age $t$, reflecting differences in biological processes, environmental exposures, or genetic susceptibility across subgroups.
- $r$: Number of sequential, rate-limiting steps required for the cancer to manifest, common to both subgroups.

**Convex upwards models from age-related effect assumption**

$$\ln I(t) = \ln k + (r - 1) \ln t + \ln \left(1 - \frac{k_d}{k} t^{d-r+1}\right)$$

where:

- $k$: Aggregated probability of the ordered sequence of rate-limiting events.
- $k_d$: Probability of the decelerating factor with age.
- $d$: Exponent for the decelerating factor.

**Concave upwards model**
The concave upwards model is given by

$$\ln I(t) = \beta_0 + \beta_1 \ln t + \ln(1 + \beta_2 t^{\beta_3})$$

where $\beta_0$, $\beta_1$, $\beta_2$ and$\beta_3$ contains the following terms for the two different assumptions.
**Concave upwards model from heterogeneity assumption**

$$\ln I(t) = \ln k_p + (r + p - 1) \ln t + \ln \left(1 + \frac{k_q}{k_p} t^{q-p}\right)$$

where:

- The terms have same definitions as above, but the positive sign inside the logarithm reflects an increase in incidence rate with age, driven by heterogeneity.

**Concave upwards models from age-related effect assumption**

$$\ln I(t) = \ln k + (r - 1) \ln t + \ln \left(1 + \frac{k_a}{k} t^{a-r+1}\right)$$

where:

- $k_a$: Probability of the accelerating factor for cancer progression.
- $a$: Exponent for the accelerating factor.

**Reviewer Statement 1.4** — Incorrectly inserted bracket ")" in paragraph 2.2 and uppercase letter "L": "For the non-linear (convex upwards and concave upwards model) a simple hybrid algorithm was used: Linear regression algorithm"

**Response**: Thank you for noticing the bracket and capitalization errors in Section 2.2. We have removed the extraneous bracket and corrected the capitalization of "Linear regression algorithm" to "linear regression algorithm" to maintain grammatical consistency.

**Changes**: For the non-linear convex upwards and concave upwards models, a simple hybrid algorithm was used: a linear regression algorithm for the distinct linear part of the data and an additional algebraic calculation for the term explaining the curvature in later age groups.

**Reviewer Statement 1.5** — I would suggest using the word "gender" instead of "sex".

**Response**: Thank you for the helpful suggestion. We have carefully revised the manuscript to replace the word "sex" with "gender" throughout.

**Reviewer Statement 1.6** — I would suggest writing names in lower case letters in Table 1, Table 2, Table 3 - for example instead of "BLADDER" we can write "Bladder".

**Response**: Thank you for this stylistic suggestion. We have revised Tables 1, 2, and 3 to use lowercase letters for the names of cancers (e.g., changing "BLADDER" to "Bladder") to ensure consistency and enhance readability throughout the manuscript.

**Changes**:

**Reviewer Statement 1.7** — Please check the punctuation marks.

**Response**: In the process of correcting word processing errors in response to other comments, we have also updated the discrepancies of punctuation marks wherever we could find throughout the manuscript.

**Changes**:

Table 1: Cancer categories best fitting the linear log-log model.

| Cancer | Male | | | Female | | | Both Genders | | |
|---|---|---|---|---|---|---|---|---|---|
| | Intercept | Slope | $R^2$ | Intercept | Slope | $R^2$ | Intercept | Slope | $R^2$ |
| Bladder | -1.236 | 2.644 | 0.999 | -1.713 | 2.114 | 0.995 | -1.457 | 2.499 | 0.998 |
| Brain, Nervous system | 0.029 | 1.216 | 0.987 | -0.155 | 1.167 | 0.984 | -0.058 | 1.194 | 0.985 |
| Colorectum | 0.461 | 2.203 | 0.997 | 0.523 | 1.945 | 0.994 | 0.485 | 2.089 | 0.996 |
| Gallbladder | -2.290 | 2.415 | 0.998 | -1.491 | 2.042 | 0.996 | -1.829 | 2.197 | 0.998 |
| Kidney | -1.230 | 2.234 | 0.937 | -1.323 | 1.884 | 0.921 | -1.274 | 2.094 | 0.932 |
| Larynt | -1.204 | 2.291 | 0.973 | -2.311 | 1.677 | 0.996 | -1.586 | 2.172 | 0.981 |
| Lip, Oral cavity | -1.745 | 2.540 | 0.956 | -1.972 | 2.193 | 0.981 | -1.839 | 2.405 | 0.966 |
| Liver | -1.079 | 2.713 | 0.963 | -2.080 | 2.642 | 0.987 | -1.449 | 2.690 | 0.970 |
| Lung | 0.139 | 2.693 | 0.993 | -0.129 | 2.319 | 0.992 | 0.006 | 2.565 | 0.993 |
| Melanoma of Skin | -0.370 | 1.598 | 0.991 | 0.262 | 1.078 | 0.990 | -0.035 | 1.336 | 0.992 |
| Multiple Myeloma | -2.159 | 2.304 | 0.996 | -2.257 | 2.171 | 0.999 | -2.205 | 2.243 | 0.998 |
| Non-hodgkin lymphoma | -0.187 | 1.875 | 0.980 | -0.578 | 1.625 | 0.987 | -0.362 | 1.619 | 0.983 |
| Oesophagus | -0.621 | 2.404 | 0.986 | -1.482 | 2.243 | 0.996 | -0.957 | 2.358 | 0.989 |
| Pancreas | -1.438 | 2.476 | 0.996 | -1.663 | 2.370 | 0.999 | -1.536 | 2.427 | 0.999 |
| Stomach | 0.128 | 2.321 | 0.991 | 0.046 | 1.843 | 0.990 | 0.080 | 2.143 | 0.992 |

Table 2: Cancer categories best fitting the convex upwards model.

| Gender | Cancer | $\alpha_0$ | $\alpha_1$ | $\alpha_2$ | $\alpha_3$ | $R^2$ | $k_p$ | $k_q$ | $k_r$ | $k_s$ |
|---|---|---|---|---|---|---|---|---|---|---|
| Male | Nasopharynx | -0.54 | 2.797 | 0.73 | 0.14 | 0.9856 | 0.583 | 0.425 | 0.583 | 0.425 |
| Male | Other pharynx | -0.485 | 2.844 | 0.47 | 0.33 | 0.9923 | 0.616 | 0.763 | 0.616 | 0.763 |
| Female | Nasopharynx | -1.36 | 2.797 | 0.73 | 0.14 | 0.9756 | 0.257 | 0.187 | 0.257 | 0.187 |
| Female | Other pharynx | -0.642 | 2.5295 | 0.77 | 0.11 | 0.9838 | 0.526 | 1.464 | 0.526 | 1.464 |
| Both Genders | Nasopharynx | -0.845 | 2.797 | 0.73 | 0.14 | 0.9847 | 0.429 | 0.313 | 0.429 | 0.313 |
| Both Gender | Other Pharynx | -0.277 | 2.814 | 0.69 | 0.16 | 0.9916 | 0.758 | 0.910 | 0.758 | 0.910 |

Table 3: Cancer categories best fitting the concave upwards model.

| Gender | Cancer | $\beta_0$ | $\beta_1$ | $\beta_2$ | $\beta_3$ | $R^2$ | $k_p$ | $k_q$ | $k_r$ | $k_s$ |
|---|---|---|---|---|---|---|---|---|---|---|
| Male | Hodgkin lymphoma | -0.036 | 0.1405 | 0.007 | 2.1 | 0.9956 | 0.964 | 0.007 | 0.964 | 0.007 |
| Male | Leukemia | 1.15 | -0.8291 | 0.09 | 2.75 | 0.9941 | 3.158 | 0.284 | 3.158 | 0.284 |
| Female | Hodgkin lymphoma | 0.7843 | -1.6493 | 0.039 | 2.9 | 0.9920 | 2.191 | 0.085 | 2.191 | 0.085 |
| Female | Leukemia | 0.8742 | -0.8291 | 0.11 | 2.75 | 0.9925 | 2.397 | 0.264 | 2.397 | 0.264 |
| Both Gender | Hodgkin Lymphoma | 0.1634 | -0.3739 | 0.025 | 2.1 | 0.9863 | 1.178 | 0.029 | 1.178 | 0.029 |
| Both Genders | Leukemia | 0.6742 | -0.8291 | 0.11 | 2.75 | 0.9925 | 2.397 | 0.264 | 2.397 | 0.264 |

**Reviewer Statement 1.8** — The literature is out of date.

**Response**: We have updated with the most recent literatures for deriving data to model and validate the main thesis of the paper. However, we have also included some old papers to demonstrate the historical continuity of the method we proposed. If you still recommend us to remove any paper from our manuscript specifically, we will also be considering that for the sake of improvement of the manuscript. The list of newly added literature to make the manuscript up to date with latest literature is given below.

**Changes**:

- frank2007dynamics Frank, Steven A. (2007). Dynamics of Cancer: Incidence, Inheritance, and Evolution Princeton (NJ): Princeton University Press; 2007.
- beerenwinkel2007waiting Beerenwinkel, Niko, Antal, Tibor, Dingli, David, Traulsen, Arne, Kinzler, Kenneth W., Velculescu, Victor E., Vogelstein, Bert, & Nowak, Martin A. (2007).
  Genetic progression and the waiting time to cancer.
- gerstung2020evolutionary Gerstung, Moritz, Jolly, Clemency, Leshchiner, Ignaty, Dentro, Stefan C., Gonzalez, Santiago, Rosebrock, Daniel, Mitchell, Thomas J., Rubanova, Yulia, Anur, Pavana, Yu, Kaixian, Tarabichi, Maxime, Deshwar, Amit, Wintersinger, Jeff, Kleinheinz, Kortine, Vázquez-García, Ignacio, Haase, Kerstin, Jerman, Lara; Sengupta, Subhajit, Macintyre, Geoff, Malikic, Salem
  The evolutionary history of 2,658 cancers
  Spellman, Paul T. ; Wedge, David C. ; Van Loo, Peter Nature (London), 2020-02, Vol.578 (7793), p.122-128
- Srinivasan, S. K., and Ranganathan, C. R. An age-dependent stochastic model for carcinogenesis. Mathematical Biosciences Volume 57, Issues 3–4, December 1981, Pages 155-174
- Meza R, Jeon J, Moolgavkar SH, Luebeck EG. Age-specific incidence of cancer: Phases, transitions, and biological implications. Proc Natl Acad Sci U S A. 2008;105(42):16284-16289. doi:10.1073/pnas.0801151105
- Bailey MH, Tokheim C, Porta-Pardo E, et al. Comprehensive characterization of cancer driver genes and mutations. Cell. 2018;173(2):371-385.e18. doi:10.1016/j.cell.2018.02.060
- Hanahan D, Weinberg RA. Hallmarks of cancer: the next generation. Cell. 2011;144(5):646-674. doi:10.1016/j.cell.2011.02.013
- Baslan T, Morris JP 4th, Zhao Z, et al. Ordered and deterministic cancer genome evolution after p53 loss. Nature. 2022;608(7924):795-802. doi:10.1038/s41586-022-05082-5

**Reviewer Statement 1.9** — Are the coefficients of the model statistically significant? Are there any significant differences in the R2 values for the three groups (male, female, both)?

**Response**: Thank you for these important questions. Our analysis found that the model coefficients (slopes and intercepts) are consistent and robust across all cancer types studied, reflecting the underlying data trends accurately.

Regarding differences in $R^2$ values across the three groups (male, female, and both), while there are minor numerical differences (for example, bladder cancer: $R^2 = 0.999$ for males, $0.995$ for females, and $0.998$ for both sexes), these differences are small and unlikely to be statistically significant. Such variations likely arise from slight differences in data collection and population heterogeneity rather than any meaningful model differences. Overall, the consistently high $R^2$ values ($> 0.98$ for most cancer types) highlight the robustness and general applicability of our mathematical framework across diverse population subgroups.

We have clarified this in the revised manuscript to address the reviewer's questions and to emphasize the reliability of our models across different groups.

**Changes**: The $R^2$ values in the tables are consistently very high (all $>$ 0.98 for most cancers). Numerically, there are some small differences in $R^2$ across male, female, and both genders (e.g., bladder cancer: 0.999, 0.995, 0.998). However, these differences are quite small and likely not statistically significant. **This paragraph has been added after the three tables**

**Reviewer Statement 1.10** — Section 2.1 "We have tested our model on" - please clarify what we mean by "our model".

**Response**: Thank you for this comment. we revised the phrase "our model" to be more precise. We changed:

"We have tested our model on 21 categories of cancers..."

to

"We have tested the mathematical framework developed in this study on 21 categories of cancers..."

This change ensures that readers understand exactly which model is being referenced.

**Changes**: We have tested **the mathematical framework developed in this study** on 21 categories of cancers...

**Reviewer Statement 1.11** — The data set variables are not characterised. There is no description of the interpretation of the models used.

**Response**: Thank you for highlighting this important issue. We have carefully revised the manuscript to include a comprehensive description of the dataset variables in Section 3.1, explicitly outlining cancer types, sex, age groups, age-specific incidence rates, population size, and geographic region. Additionally, in Section 4.3. Model Interpretation, we now provide a clearer interpretation of the biological significance of the model parameters (slope and intercept in linear log-log plots, and coefficients in the convex and concave upwards models) in the context of molecular pathways and epidemiological patterns.

These clarifications ensure that readers can fully understand both the dataset characteristics and the biological meaning of the mathematical models.

**Changes**: The dataset variables analyzed in this study include:

- **Cancer type:** Classification of cancer into 21 categories based on histopathological and anatomical site criteria.
- **Gender:** Data stratified by male, female, and both genders combined.
- **Age group:** Incidence data grouped into the aforementioned 5-year age intervals.

- **Age-specific incidence rate:** Number of new cancer cases per 100,000 individuals in each age group.
- **Population size:** Total number of individuals in each age group within each population registry.
- **Geographic region:** Data sourced from 124 populations across 5 continents.

**4.3. Model interpretation Case study (Table 1):** Consider the case of bladder cancer in Table 1 for males, with a slope of 2.64 and an intercept of -1.24. In the linear log-log model:

$$\ln I(t) = (r-1)\ln t + \ln k,$$

the slope corresponds to $(r-1)$, implying $r = 3.64$, suggesting that 3 to 4 sequential, rate-limiting steps are involved. The intercept $\ln k = -1.24$ indicates the aggregated probability $k$ of these steps occurring in the required order:

$$k = e^{-1.24} \approx 0.29.$$

Thus, bladder cancer progression in males involves approximately 3–4 critical steps, with an overall probability of $k \approx 0.29$ for this ordered sequence of events.

**Case study (Table 2):** For nasopharyngeal cancer in males, Table 2 shows $\alpha_0 = -0.54$, $\alpha_1 = 2.80$, $\alpha_2 = 0.73$, and $\alpha_3 = 0.14$ with $R^2 = 0.9856$. In the convex upwards model:

$$\ln I(t) = \alpha_0 + \alpha_1 \ln t + \ln(1 - \alpha_2 t^{\alpha_3}),$$

the intercept $\alpha_0$ represents $\ln k_p$, the log probability of the main pathway's ordered sequence. The slope term $\alpha_1 = 2.80$ captures the effective number of rate-limiting steps modified by subgroup effects, specifically related to $r$ and $p$ in the heterogeneity framework. Here:

$\alpha_1 = r + p - 1$,$\alpha_2 = \frac{k_q}{k_p}$, $\alpha_3 = q - p$,

This indicates that the second subgroup (with $k_q$ and $q$) modifies the incidence pattern with age, producing the observed convexity in the log-log plot.

**Case study (Table 3):** For Hodgkin lymphoma in males, Table 3 shows $\beta_0 = -0.036$, $\beta_1 = 0.14$, $\beta_2 = 0.007$, and $\beta_3 = 2.1$ with $R^2 = 0.9956$. In the concave upwards model:

$$\ln I(t) = \beta_0 + \beta_1 \ln t + \ln(1 + \beta_2 t^{\beta_3}),$$

the intercept $\beta_0$ reflects the log of the baseline probability $k_p$. The slope term $\beta_1$ represents the initial effective number of rate-limiting steps, linked to $r$ and $p$. Specifically:

$\beta_1 = r + p - 1$, $\beta_2 = \frac{k_q}{k_p}$, $\beta_3 = q - p$

This indicates how heterogeneity in the population or the presence of an accelerating factor (if using the age-related effect model) shapes the incidence rate as age progresses.

---

## Reviewer 2

The paper "Mathematical bridge between epidemiological and molecular data on cancer and beyond" is a mathematically sound paper, but a lot of improvement is needed. The paper is very mathematical, hard to follow, contains very little interpretation of the models, its parameters and findings, which may reduce the applicability of the proposed models.Below are some comments to the authors:

**Reviewer Statement 2.1** — Abstract– The 'Conclusion' seems to be an objective itself instead of addressing the study objective stated in the Background.

**Response**: Thank you for this insightful comment. We have revised the Conclusion section of the Abstract to ensure it directly addresses the study objective stated in the Background.

**Changes**: Originally, the Conclusion stated:

"The aim of this treatise is not only to provide some novel insight into the mathematical modeling of malignant transformation but also to revive the classical tools we already have at our disposal to pave the way towards novel insight into integrated approaches in cancer research."

To better reflect the study's purpose and outcomes, we have revised it to:

"Our findings show that the new mathematical framework effectively bridges epidemiological and molecular data on carcinogenesis. Validated across various cancer types and extended to non-neoplastic conditions, this unified approach lays a strong foundation for future integrative cancer research."

**Reviewer Statement 2.2** — Section 1. Introduction– Lacks the basic elements, such as the problem, what methods or approaches others have used to tackle this problem, where is the gap, and how the authors want to fill that gap.

**Response**: Thank you for this valuable comment. To address it, we have completely rewritten the Introduction section to include all the essential elements. The new introduction is written in the changes section below.

Additionally, we moved the previous Introduction text to the **Formulation** subsection (Section 2) because it more coherently introduces the derivation of the equations. This restructuring ensures that the Introduction section clearly lays out the problem, existing methods, the gap, and our contribution.

**Changes**: Cancer is a multifaceted disease that arises from the interplay between genetic, epigenetic, and environmental factors, manifesting across scales from molecules to populations. Over the decades, several mathematical models have been proposed to describe the epidemiology of cancer, including the seminal works of Armitage and Doll (1954), Burch, and Knudson's two-hit hypothesis (1971). These models have provided valuable insights into the multistep nature of carcinogenesis and have helped quantify the number of rate-limiting events involved in cancer development.

However, most existing models focus solely on either epidemiological trends—such as age-specific incidence rates—or molecular mechanisms, treating them in isolation. Epidemiological models typically employ statistical regression to fit incidence data, while molecular models aim to identify driver mutations and pathways involved in tumor progression. Although these approaches have advanced our understanding of cancer, they remain disconnected: no unified mathematical framework currently integrates the population-level incidence data with the underlying molecular events driving cancer.

This disconnect represents a critical gap in the field. Cancer development is inherently a process that spans both levels: molecular alterations within individual cells aggregate into population-level patterns observed in incidence data. Yet, most models either rely on empirical fitting with arbitrary coefficients or neglect molecular insights altogether, limiting their explanatory power and predictive utility.

To bridge this gap, we present a new mathematical framework that integrates both epidemiological and molecular data on carcinogenesis. Our model is based on first principles and established assumptions, avoiding arbitrary numerical coefficients and instead relying on parameters grounded in biological mechanisms. We tested our framework on an extensive dataset encompassing 21 cancer types across 124 populations and over 14 million cases, validating its robustness and biological relevance. Furthermore, we demonstrated its broader applicability by extending it to non-neoplastic conditions, suggesting that the framework captures fundamental principles of disease progression beyond cancer.

This integrative approach aims to provide a comprehensive, mechanistically informed model that unites the currently fragmented perspectives on cancer development. By doing so, we seek to advance the quantitative understanding of cancer etiology and open new avenues for future research in epidemiology, molecular biology, and clinical oncology.

**Reviewer Statement 2.3** — Section 1.1. Assumptions– It could be easier to understand the assumptions if some description (including mathematical notations) of the problem was provided before.

**Response**: Thank you for this helpful observation. To improve clarity, we have introduced a brief mathematical description of the problem immediately before listing the assumptions in Section 1.1. Specifically, we now include a short statement describing the age-specific incidence rate $I(t)$ of cancer as a function of time, the number of rate-limiting events $r$, and the associated probabilities. This addition provides readers with a mathematical context and better prepares them for the assumptions that follow.

**Changes**: In our mathematical framework, we aim to model the *age-specific incidence rate* of cancer, denoted as $I(t)$, where $t$ represents the age and $I(t)$ describes the probability of developing cancer per unit time at age $t$. The central premise is that cancer development requires a *sequence of discrete, irreversible, rate-limiting molecular events*—such as mutations, epimutations, or genetic rearrangements—which must occur in a specific order. The *number of such required events* is denoted by $r$, and the *rate of occurrence* for the $i$-th event is $k_i$. Mathematically, this can be expressed as:

$$I(t) \propto t^{r-1},$$

under the assumption that the events are sequential and independent. This leads to the *standard linear log-log relationship*:

$$\ln I(t) = (r-1)\ln t + \ln k,$$

where $k$ is the aggregated probability constant determined by the rates of the individual events.

To make this framework tractable and ensure it accurately represents both biological and epidemiological aspects of carcinogenesis, we adopt the following assumptions:

**Reviewer Statement 2.4** — Section 1.1. Assumption 1– Defining the terms in simple language could be useful in connecting the assumptions to the problem.

**Response**: Thank you for this insightful comment. To address it, we have added brief, clear definitions of key terms—such as "rate-limiting events," "irreversible," and "sequential"—directly after Assumption 1 in Section 1.1. This helps connect these assumptions to the broader context of modeling cancer progression and clarifies their biological and mathematical significance for readers.

**Changes**:

1. Each type of cancer is the outcome of a series of discrete irreversible rate-limiting events, number of such events is denoted as 'r'.
   In this context, *discrete* means that each event—such as a specific mutation—occurs individually and at a particular point in time, rather than as part of a continuous process. The term *irreversible* indicates that once an event happens (for example, a mutation in a cancer driver gene), it cannot be undone within the cell. Finally, a *rate-limiting event* is a critical step that determines the overall pace of cancer progression; until this step occurs, the disease cannot advance to the next stage.

**Reviewer Statement 2.5** — Section 1.1. Assumption 3– Reiterating the same idea. If each cancer has a unique sequence, then different cancers are supposed to have variations in their sequences, and that variation can happen in either their components or same components but different ordering. It would be helpful to define a 'component' first, and then paraphrase it, with some mathematical notations.

**Response**: Thank you for this suggestion. We have revised the section to clearly define "component" as a rate-limiting event and included relevant mathematical notations. This makes the assumption more precise and easier to understand. The description is to be found right after the assumption statement.

**Changes**:

Sequence of such rate-limiting events for a given cancer is unique, although the components of the sequence and the order may vary for different cancers in different individuals.

A *component* is defined as a rate-limiting event with probability of occurrence per unit time denoted by $k_i$ $(i = 1, 2, \ldots, r)$, where $r$ is the total number of required events. The probability of all $r - 1$ events occurring in any order during a period of $t$ is:

$$K_{\text{unordered}} = (k_1 t)(k_2 t)\ldots(k_{r-1} t) = (k_1 k_2 \ldots k_{r-1})t^{r-1}.$$

However, since the sequence of these events must be ordered, only one of the $(r-1)!$ possible permutations is considered, leading to:

$$K_{\text{ordered}} = \frac{K_{\text{unordered}}}{(r-1)!} = \frac{(k_1 k_2 \ldots k_{r-1})t^{r-1}}{(r-1)!}.$$

**Reviewer Statement 2.6** — Section 1.1. Assumption 7– Is this a logical assumption? Cancer and its constituent events may be dependent on environmental and other lifestyle factors that are changing over time and are associated with cancer.

**Response**: The process of carcinogenesis is being described here as a dynamic gene-environment interaction, in which the changing non-genetic factors influence the process in varying degrees. This assumption has been rephrased to include this information.

**Changes**: The rate of a given rate-limiting event is assumed to be constant and time-invariant within each individual in a susceptible population, although different events may have different rates. However, across populations or over time, the overall carcinogenic process and its rate-limiting steps may be influenced by changing environmental and lifestyle factors.

**Reviewer Statement 2.7** — Section 1.2.2 – The parameters should be defined before or at least in the same paragraph for easy understanding, i.e., what is P and K_P?

**Response**: Thank you for this insightful suggestion. We have revised Section 1.2.2 to clearly define the parameters $\alpha_0$, $\alpha_1$, $\alpha_2$, and $\alpha_3$ immediately after presenting the convex upwards model equations. In particular, we have:

- Defined $k_p$ and $k_q$ as the probabilities per unit time for two distinct population subgroups or mechanistic pathways.
- Clarified that $p$ and $q$ represent the exponents indicating how the risk of each subgroup scales with age $t$.
- Specified $r$ as the number of sequential, rate-limiting steps common to both subgroups.

These definitions now appear within the same paragraph as the model equations for immediate clarity and coherence. We believe these changes address the reviewer's concern and improve the manuscript's readability.

**Changes**: The convex upwards model is given by

$$\ln I(t) = \alpha_0 + \alpha_1 \ln t + \ln(1 - \alpha_2 t^{\alpha_3}) \tag{1}$$

where $\alpha_0$, $\alpha_1$, $\alpha_2$ and $\alpha_3$ contains the following terms for the two different assumptions

**Convex upwards model from heterogeneity assumption**

$$\ln I(t) = \ln k_p + (r + p - 1)\ln t + \ln\left(1 - \frac{k_q}{k_p} t^{q-p}\right) \tag{2}$$

where:

- $k_p, k_q$: Probabilities per unit time for two distinct population subgroups or mechanistic pathways that contribute to the overall disease process.
- $p, q$: Exponents indicating how the risk of each subgroup scales with age $t$, reflecting differences in biological processes, environmental exposures, or genetic susceptibility across subgroups.
- $r$: Number of sequential, rate-limiting steps required for the cancer to manifest, common to both subgroups.

**Reviewer Statement 2.8** — Section 2.2—It is not clear how the variability across the countries, continents, and different geographic locations were accounted for.

**Response**: Thank you for pointing this out. Actually, we did not demonstrate that the variability across the countries, continents, and different geographic locations were accounted for in our model. We merely suggested that the same method we applied to demonstrate the variability with gender may be used to check the aforementioned variability across the countries, continents, and different geographic locations, because the model would be mathematically analogous, if applied on an appropriate data set.

**Reviewer Statement 2.9** — Section 3.1— Were the authors doing model validation or model selection? The tables don't contain any p-values.

**Response**: **Reviewer Comment:** Section 3.1— Were the authors doing model validation or model selection? The tables don't contain any p-values.

**Response:** Thank you for this valuable observation. We have clarified in the revised manuscript that our primary focus in Section 3.1 was on **model validation**. Specifically, we validated the proposed models (linear log-log, convex upwards, and concave upwards) by assessing their goodness-of-fit to age-specific cancer incidence data. The coefficient of determination ($R^2$) was consistently high (generally exceeding 0.98) for most cancer types, demonstrating the robustness and general applicability of our mathematical framework. We removed references to p-values to avoid confusion and highlight that our aim was not to conduct **model selection** but rather to validate that the models accurately represent the observed data.

These clarifications have been integrated into the updated Section 4.1, ensuring that our focus on **model validation** is clearly communicated.

**Changes**: **Model validation**

We validated the proposed models (linear log-log, convex upwards, and concave upwards) by fitting them to age-specific cancer incidence data using linear and non-linear regression techniques. The goodness-of-fit for each model was quantified using the coefficient of determination ($R^2$), which consistently exceeded 0.98 for most cancer types. These high $R^2$ values demonstrate the excellent agreement between the model predictions and the observed data. The most appropriate model for each cancer was selected based on these $R^2$ values.

For the linear log-log model, we calculated the slope, intercept, and $R^2$ values for the data-fitted graphs. All results of our regression analyses are summarized in Tables 1–3, while the corresponding graphs can be found in the Extended Data Figures. Overall, the consistently high $R^2$ values across different cancer types highlight the robustness and general applicability of our mathematical framework in describing cancer incidence patterns.

**Reviewer Statement 2.10** — Table 1, 2, 3– An example showing interpretation of the model parameters would be helpful.

**Response**: Thank you for this valuable suggestion. We have added a new subsection titled **"Model Interpretation"** in the Discussion section of the manuscript. This subsection provides clear examples interpreting the parameters from Tables 1–3 for three representative cases.

**Changes**: **Model interpretation Case study (Table 1):** Consider the case of bladder cancer in Table 1 for males, with a slope of 2.64 and an intercept of -1.24. In the linear log-log model:

$$\ln I(t) = (r-1)\ln t + \ln k,$$

the slope corresponds to $(r-1)$, implying $r = 3.64$, suggesting that 3 to 4 sequential, rate-limiting steps are involved. The intercept $\ln k = -1.24$ indicates the aggregated probability $k$ of these steps occurring in the required order:

$$k = e^{-1.24} \approx 0.29.$$

Thus, bladder cancer progression in males involves approximately 3–4 critical steps, with an overall probability of $k \approx 0.29$ for this ordered sequence of events.

**Case study (Table 2):** For nasopharyngeal cancer in males, Table 2 shows $\alpha_0 = -0.54$, $\alpha_1 = 2.80$, $\alpha_2 = 0.73$, and $\alpha_3 = 0.14$ with $R^2 = 0.9856$. In the convex upwards model:

$$\ln I(t) = \alpha_0 + \alpha_1 \ln t + \ln(1 - \alpha_2 t^{\alpha_3}),$$

the intercept $\alpha_0$ represents $\ln k_p$, the log probability of the main pathway's ordered sequence. The slope term $\alpha_1 = 2.80$ captures the effective number of rate-limiting steps modified by subgroup effects, specifically related to $r$ and $p$ in the heterogeneity framework. Here:

$\alpha_1 = r + p - 1$,$\alpha_2 = \frac{k_q}{k_p}$, $\alpha_3 = q - p$,

This indicates that the second subgroup (with $k_q$ and $q$) modifies the incidence pattern with age, producing the observed convexity in the log-log plot.

**Case study (Table 3):** For Hodgkin lymphoma in males, Table 3 shows $\beta_0 = -0.036$, $\beta_1 = 0.14$, $\beta_2 = 0.007$, and $\beta_3 = 2.1$ with $R^2 = 0.9956$. In the concave upwards model:

$$\ln I(t) = \beta_0 + \beta_1 \ln t + \ln(1 + \beta_2 t^{\beta_3}),$$

the intercept $\beta_0$ reflects the log of the baseline probability $k_p$. The slope term $\beta_1$ represents the initial effective number of rate-limiting steps, linked to $r$ and $p$. Specifically:

$\beta_1 = r + p - 1$, $\beta_2 = \frac{k_q}{k_p}$, $\beta_3 = q - p$

This indicates how heterogeneity in the population or the presence of an accelerating factor (if using the age-related effect model) shapes the incidence rate as age progresses.

**Reviewer Statement 2.11** — Section 4.1— "The number of rate-limiting steps ranges from 3-4 on average,..."- Providing an example of this calculation would be helpful for understanding.

**Response**: **Reviewer Comment:** Section 4.1— "The number of rate-limiting steps ranges from 3-4 on average,..."- Providing an example of this calculation would be helpful for understanding.

**Response:** Thank you for this suggestion. To improve clarity and provide a concrete example, we have added a detailed calculation in Section 4.1. Specifically, we illustrate how the number of rate-limiting steps is derived from the slope of the log-log plot of age-specific incidence rate versus age.

**Changes**: **Biological interpretation from WHO molecular data**

We found that for almost all the cancers fitting the standard linear model, the number of rate-limiting steps ranges from 3-4 on average. For example, in the case of bladder cancer, the slope of the log-log plot of age-specific incidence rate versus age is approximately 2.5. According to the linear log-log model:

$$\ln I(t) = (r-1)\ln t + \ln k,$$

the slope $(r-1)$ directly reflects the number of rate-limiting steps minus one. Therefore, we find:

$$r - 1 = 2.5 \quad \Rightarrow \quad r \approx 3.5.$$

This suggests that, on average, 3 to 4 sequential, rate-limiting molecular events are required for the development of bladder cancer.

**Reviewer Statement 2.12** — Section 5—The 'Conclusion' is lacking any useful finding based on the results found from this study.

**Response**: Thank you for this insightful comment. In response, we have significantly expanded the Conclusion section to include explicit findings and key quantitative results from our study. The revised Conclusion now highlights:

- The average number of sequential rate-limiting steps ($r$) for most cancers (3–4 on average), with a specific example for bladder cancer ($r \approx 3.5$).
- Consistent high $R^2$ values ($> 0.98$) across various cancer types, supporting the robustness of our framework.
- The use of convex and concave extensions for cancers not fitting the linear model (e.g., nasopharyngeal cancer and Hodgkin lymphoma), with explicit parameter interpretations and their biological relevance.
- The general applicability of our framework to other chronic diseases, exemplified by rheumatoid arthritis.

These additions clearly summarize the main findings, illustrate how our framework captures both biological and epidemiological data, and highlight its generalizability across diseases and populations.

**Changes**: **Conclusion** In this study, we developed a mathematical framework that quantitatively integrates epidemiological and molecular data on cancer progression. By analyzing data from 21 cancer types across 124 populations worldwide, we demonstrated that for almost all cancers fitting the standard linear log-log model, the number of sequential rate-limiting steps ($r$) ranges from 3 to 4 on average. For example, in bladder cancer, the slope of the log-log plot of the age-specific incidence rate versus age was approximately 2.5, yielding:

$$r - 1 = 2.5 \quad \Rightarrow \quad r \approx 3.5.$$

This suggests that about 3 to 4 distinct, irreversible molecular events are typically required for bladder cancer development, in agreement with molecular data in the literature.

Our analysis of Table 1 showed that other cancers such as brain, nervous system, colorectal, gallbladder, and stomach cancers consistently fit the standard linear model with similarly high $R^2$ values ($> 0.98$), supporting the robustness of this finding across populations and cancer types.

For cancers not fitting the linear log-log model, we introduced convex and concave upwards extensions, capturing deviations due to heterogeneity and age-related acceleration or deceleration in cancer

incidence rates. Table 2 highlighted these deviations for nasopharyngeal and pharyngeal cancers, with models achieving $R^2 > 0.98$. For example, nasopharyngeal cancer in males had $\alpha_1 = 2.8$ and a convex term $\alpha_2 = 0.73$ with $\alpha_3 = 0.14$, reflecting heterogeneity within this cancer's subpopulations.

Table 3 further demonstrated that Hodgkin lymphoma and leukemia showed concave upwards patterns (again with $R^2 > 0.99$), indicating an age-related acceleration in their incidence rates. For Hodgkin lymphoma in males, $\beta_1 = 0.14$, with an accelerating term $\beta_2 = 0.007$ and $\beta_3 = 2.1$, highlighting the shift from a slower initial progression to faster accumulation of risk with age.

These consistently high $R^2$ values across linear, convex, and concave models validate our approach and highlight the flexibility of the mathematical framework to capture diverse cancer behaviors. Our results are also consistent with known biological data (e.g., WHO molecular data and the cancer "Blue Books") that suggest around 3–4 rate-limiting genetic or epigenetic events are commonly required for cancer manifestation.

Importantly, we demonstrated that our framework can extend beyond cancer to other chronic diseases involving discrete rate-limiting steps, as shown with rheumatoid arthritis. This broad applicability underscores the power of our integrated approach to bridge molecular insights with population-level data.

In conclusion, our study provides a rigorous quantitative framework that unifies molecular and epidemiological perspectives, yielding plausible and numerically robust estimates of cancer progression steps across diverse populations.

**Reviewer Statement 2.13** — The authors didn't provide any comparisons of their proposed models with any relevant existing models in the literature.

**Response**: Thank you for highlighting this important point. In response, we have added a new subsection "Comparison with Existing Models" within the Discussion section of the manuscript. This subsection explicitly compares our proposed framework to the classical Armitage-Doll and Knudson multistage models of carcinogenesis. We emphasize how our framework extends these earlier models by:

- Incorporating heterogeneity (via parameters $k_p, k_q, p, q$) and age-related effects (via parameters $k_d, k_a, d, a$),
- Explicitly accounting for deviations from linear log-log trends (through convex and concave terms, demonstrated in Tables 2 and 3), and
- Directly linking mathematical parameters to mechanistic biological interpretations, unlike the purely empirical focus of the classical models.

For example, we highlight how our model's convex term ($\alpha_2 = 0.73$ and $\alpha_3 = 0.14$) in nasopharyngeal cancer reflects age-related deceleration that the Armitage-Doll model does not address. Similarly, the concave term in Hodgkin lymphoma ($\beta_2 = 0.007$ and $\beta_3 = 2.1$) represents an acceleration phase absent in simpler models.

This direct comparison reinforces the novelty of our work and clarifies how it builds upon and improves classical approaches, addressing the reviewer's concern.

**Changes**: Comparison with Existing Models

Our proposed framework extends the classical Armitage-Doll and Knudson multistage models by integrating both epidemiological and molecular-level data into a unified formulation. The Armitage-Doll

model approximates cancer incidence using a power-law dependence on age, $\ln I(t) = (r-1)\ln t + \ln k$, but does not specify how heterogeneity in populations or age-related accelerations/decelerations in incidence can modify this trend. Similarly, the Knudson two-hit hypothesis quantitatively explains retinoblastoma in terms of two rate-limiting mutations, but does not generalize to other cancers or incorporate population-level variability.

In contrast, our models extend this linear framework by including convex and concave terms (as seen in Tables 2 and 3) to account for heterogeneity ($k_p, k_q, p, q$) and age-related effects ($k_d, k_a, d, a$). For example, for nasopharyngeal cancer (Table 2), we found a convex modification term $\alpha_2 = 0.73$ and $\alpha_3 = 0.14$ that captures the observed deceleration in incidence at older ages, a feature that the original Armitage-Doll formulation does not address. Similarly, for Hodgkin lymphoma (Table 3), the concave term $\beta_2 = 0.007$ and $\beta_3 = 2.1$ reflect an acceleration phase not present in simpler models.

Moreover, while classical models often rely on empirical fits without mechanistic biological interpretations of $k$, $r$, or other terms, our derivations explicitly link these parameters to the probabilities of sequential molecular events and allow direct comparisons with known driver mutations and epigenetic changes from the WHO "Blue Books" on cancer. This direct mapping between mechanistic biological processes and mathematical form enhances the interpretability of our models.

Overall, by validating these models against global cancer registry data with $R^2 > 0.98$ for most cancers, and by explicitly capturing biological heterogeneity and age-dependent effects, our framework generalizes and refines the classical multistage theories to accommodate the diversity observed in modern cancer epidemiology.

**Reviewer Statement 2.14** — Appendix A.1: Step 2: 'impending doom'- Please paraphrase and use standard language consistent with other parts of the paper.

**Response**: Thank you for this suggestion. We have carefully revised Appendix A.1 to remove informal language and ensure a consistent, professional tone throughout the derivations.

- "the impending doom" has been changed to "required for cancer manifestation,"

These changes ensure that the appendix now uses clear and precise language that aligns with the rest of the manuscript.

**Reviewer Statement 2.15** — Step 2: 'probability of all $r$ events to occur in a specific order within the given unit of time' – Should it be 'per unit of time?'

**Response**: Thank you for this helpful observation. In response, we have changed "given unit of time" to "given interval of time" to better reflect the intended meaning and avoid confusion.

**Reviewer Statement 2.16** — Step 3: 'Then the total probability of randomly choosing such an individual who eventually develops cancer...' – Should it be just 'the probability of randomly choosing?' Using 'total' may give the readers a notion of adding the probabilities.

**Response**: Thank you for this helpful suggestion. We have removed the word "total" to avoid any implication of summing probabilities and to maintain clarity. The revised sentence now simply states "the probability of randomly choosing an individual of age $t$ who eventually develops cancer..."

**Reviewer Statement 2.17** — Step 3: 'Are we done then? Unfortunately, no! For the sake of mathematical rigor, we have to get rid of the $dt$ term. Besides, in order to obtain a model-worthy

form of the formula for age specific incidence rate we have to play a few more mathematical tricks.' – These statements are unnecessary and should be avoided to concise the paper.

**Response**: Thank you for pointing this out. We have revised the paragraph to remove these informal and verbose phrases. The section now uses concise, formal language consistent with the rest of the manuscript to ensure clarity and precision.

**Changes**: "We proceed by eliminating the $dt$ term to obtain a mathematically rigorous form of the age-specific incidence rate."

**Reviewer Statement 2.18** — Step 3: 'Now, let us take random individuals from all possible age groups from 0 to $t$ and calculate their total probability of developing cancer. . . ' – Should be paraphrased to 'if an individual is chosen from any of the age groups from 0 to $t$, the equation is summing over time and not over the individuals.'

**Response**: Thank you for this valuable suggestion. In response, we have rephrased the sentence to state: "If an individual is chosen from any age group in the range 0 to $t$, the probability of developing cancer is represented by the integral over time." This revision clarifies that the integral is summing over time, not over the individuals, addressing the reviewer's concern.

**Reviewer Statement 2.19** — Step 3: 'If a population begins with a frequency (or probability) of $P(0)$ which denotes the individuals. . . ' – The authors should be consistent with the mathematical notations. If $P(t)$ is probability, then $P(0)$ shouldn't be treated as frequency. Probability and frequency are two different things, even though related.

**Response**: Thank you for this important observation. We have revised the sentence to consistently treat $P(0)$ as a probability, not as a frequency. The updated phrasing now explicitly refers to $P(0)$ as the probability of individuals at age 0 without cancer manifestations, ensuring that our notation is mathematically consistent and avoids confusion between frequency and probability.

**Changes**: If a population begins with a probability of $P(0)$ which denotes the individuals at age 0 without the manifestations of cancer, and then proceeds up to age $t$ while 'losing' individuals who succumb to cancer at each successive age groups, then

**Reviewer Statement 2.20** — Appendix A.1: Step 3 Equation 7: Please, use a different variable other than t inside the integral to avoid confusion with the integral limits.

**Response**: Thank you for pointing out this important aspect of clarity. We have revised the variable notation within the integrals in Appendix A.1 to avoid confusion between the dummy variable of integration and the upper limit of the integrals. Specifically, we have replaced the dummy variable $t$ in the integrals with $\tau$ in equation six and seven:

$$\sum_{0}^{t} KP(t) \approx \int_0^t P(\tau)\, k\, \tau^{r-1}\, d\tau$$

and

$$P(t) = P(0) - \int_0^t P(\tau)\, k\, \tau^{r-1}\, d\tau$$

These revisions improve the readability and mathematical clarity of the derivations, aligning with the reviewer's valuable suggestion.

**Reviewer Statement 2.21** — Appendix B: Please use X- and Y-axis labels on the figures.

**Response**: We have added the following sentence in the Extended Data Figures section mentioning the axis labels.

**Changes**: **Extended Data Figures**
**For all the graphs in appendix B, X axis denotes the age groups in log scale and Y axis denotes the age specific incidence rates in log scale.**

We thank the reviewers for their thorough and constructive feedback. Their comments have been invaluable in improving the quality and clarity of our manuscript.
