# Supplementary material for: Mathematical bridge between epidemiological and molecular data on cancer and beyond
Source: PLoS One. 2025 Jul 28;20(7):e0328401. doi: 10.1371/journal.pone.0328401 (PMC12303294; doi:10.1371/journal.pone.0328401)
Supplement: Attachment — Submitted filename: PONE-D-25-13487_Comments.docx [file pone.0328401.s002.docx]

The paper “Mathematical bridge between epidemiological and molecular data on cancer and beyond” is a mathematically sound paper, but a lot of improvement is needed. The paper is very mathematical, hard to follow, contains very little interpretation of the models, its parameters and findings, which may reduce the applicability of the proposed models. Below are some comments to the authors:

1. Abstract-- The ‘Conclusion’ seems to an objective itself instead of addressing the study objective stated in the Background- "Any of the models adopted in those articles so far do not account for both epidemiological and molecular levels of carcinogenesis", based on the Results.
2. Section 1. Introduction-- Lacks the basic elements, such as the problem the authors are dealing with, what methods or approaches others have used to tackle this problem, where is the gap, and how the authors want to fill that gap.
3. Section 1.1. Assumptions-- It could be easier to understand the assumptions if some description (including mathematical notations) of the problem was provided before.
4. Section 1.1. Assumption 1-- Defining the terms in simple language could be useful in connecting the assumptions to the problem. Like, what does it mean to be 'discrete', 'irreversible', and 'rate-limiting event' in the scenario of cancer.
5. Section 1.1. Assumption 3-- Reiterating the same idea. If each cancer has a unique sequence, then different cancers are supposed to have variations in their sequences, and that variation can happen in either their components or same components but different ordering. It would be helpful to define a 'component' first, and then paraphrase it, with some mathematical notations.
6. Section 1.1. Assumption 7-- Is this a logical assumption? Cancer and its constituent events may be dependent on environmental and other lifestyle factors that are changing over time and are associated with cancer.
7. Section 1.2.2 -- The parameters should be defined before or at least in the same paragraph for easy understanding, i.e., what is P and K_P?
8. Section 2.2—It is not clear how the variability across the countries, continents, and different geographic locations were accounted for?
9. Section 3.1— Were the authors doing model validation or model selection? How did you use the P-value? The tables don't contain any P-values.
10. Table 1, 2, 3-- An example showing interpretation of the model parameters would be helpful.
11. Section 4.1— “The number of rate-limiting steps ranges from 3-4 on average,…”- Providing an example of this calculation would be helpful for understanding.
12. Section 5—The ‘Conclusion’ is lacking any useful finding based on the results found from this study.
13. The authors didn’t provide any comparisons of their proposed models with any relevant existing models in the literature.
14. Appendix A.1:
    a. Step 2: ‘impending doom’- Please paraphrase and use standard language consistent with other parts of the paper.
    b. Step 2: ‘probability of all r events to occur in a specific order within the given unit of time’-- Should it be 'per unit of time?'
    c. Step 3: ‘Then the total probability of randomly choosing such an individual who eventually develops cancer…’-- Should it be just 'the probability of randomly choosing?' Using ‘total’ may give the readers a notion of adding the probabilities.
    d. Step 3: ‘Are we done then? Unfortunately, no! For the sake of mathematical rigor, we have to get rid of the dt term. Besides, in order to obtain a model-worthy form of the formula for age specific incidence rate we have to play a few more mathematical tricks.’-- These statements are unnecessary and should be avoided to concise the paper.
    e. Step 3: ‘Now, let us take random individuals from all possible age groups from 0 to t and calculate their total probability of developing cancer…’-- Should be paraphrased to 'if an individual is chosen from any of the age groups from 0 to t,", the equation is summing over time and not over the individuals.
    f. Step 3: ‘. If a population begins with a frequency (or probability) of P(0) which denotes the individuals…’-- The authors should be consistent with the mathematical notations. If P(t) is probability, then P(0) shouldn't be treated as frequency. Probability and frequency are two different things, even though related.
    g. Step 3 Equation 7: Please, use a different variable other than 't' inside the integral to avoid confusion with the integral limits.
15. Appendix B: Please use X- and Y-axis labels on the figures so that the readers can easily understand what is being displayed in the graphs immediately rather than going through the captions.
